# With a new United Nations resolution on water, sanitation, hygiene, electricity, and waste in healthcare facilities, it is time for a logical framing and consistent vocabulary

**DOI:** 10.1371/journal.pstr.0000153

**Published:** 2025-01-03

**Authors:** Darcy M. Anderson, Jamie Bartram, Lucy K. Tantum, Argaw Ambelu, Mary Eyram Ashinyo, Holystone Kafanikhale, Ryan Cronk

**Affiliations:** 1 The Water Institute, Gillings School of Global Public Health, University of North Carolina at Chapel Hill, Chapel Hill, North Carolina, United States of America; 2 School of Civil Engineering, University of Leeds, Leeds, United Kingdom; 3 Division of Water and Health, Ethiopian Institute of Water Resources, Addis Ababa University, Addis Ababa, Ethiopia; 4 Department of Quality Assurance, Institutional Care Division, Ghana Health Service Headquarters, Accra, Ghana; 5 Department of Maternal and Child Health, Gillings School of Global Public Health, University of North Carolina, Chapel Hill, North Carolina, United States of America; 6 Malawi Ministry of Health and Population, Lilongwe, Malawi

On December 18, 2023, the United Nations (UN) General Assembly adopted resolution A/78/130, “Sustainable, safe and universal water, sanitation, hygiene, waste, and electricity services in health-care facilities,” which recognizes the importance of these services for the health and safety of patients and healthcare workers and aims to advance progress toward universal access [1]. This resolution is an important advocacy tool for safe healthcare systems and facilities. It advances decades of guidelines and policies from the World Health Organization (WHO), the United Nations Children’s Fund (UNICEF), and country governments.

Fulfilling the resolution’s goals requires multi-sectoral coordination and communication. However, unclear framing and a lack of shared vocabulary hinder efforts to coordinate policy, programming, and advocacy. Different sectors and stakeholders conceptualize and define the issue differently, leading to uncoordinated and duplicative efforts and neglect of some problems. For example, medical and environmental engineering professionals both use the term “hygiene” but with different meanings. There are some areas of overlap around handwashing but gaps where behaviors of non-clinical personnel (e.g., cleaners and waste handlers) are neglected.

Addressing this requires a controlled vocabulary (i.e., an authoritative term set with broad consensus among stakeholders regarding definitions [2]). Controlled vocabulary provides a common language, enhances communication, coordination, and education, and supports evidence-based decision making. We argue that environmental health in healthcare facilities currently lacks—but would benefit from—controlled vocabulary. We propose “environmental health services” as a better descriptor for capturing the services necessary to provide a safe, well-functioning healthcare environment. We describe the history of existing vocabulary, discuss shortcomings, and propose next steps.

Since 2000, the vocabulary used to describe the healthcare environment has evolved. Fig 1 shows the changes in vocabulary used to describe environmental health services, beginning with the Millennium Development Goals in 2000. The issue is often framed as “WASH” (water, sanitation, and hygiene), a term initially introduced by the Water Supply and Sanitation Collaborative Council for advocacy efforts describing household water and sanitation services. The WHO/UNICEF Joint Monitoring Programme for Water Supply, Sanitation, and Hygiene (JMP) later used “WASH” to describe water and sanitation services monitored for the Millennium Development Goals. When the Sustainable Development Goals (SDGs) expanded targets for “universal” access to WASH “for all,” the JMP interpreted this to include healthcare settings and incorporated cleaning and waste management indicators, despite their exclusion in the SDG nor conventionally understood as WASH components [3]. Some now understand the term implicitly to include cleaning and waste management, while others intend its narrower original scope.

Inconsistency in tools and guidelines contributes to confusion. In 2008, the WHO publication “Essential Environmental Health Standards in Health Care” defined the problem broadly, more comprehensively incorporating diverse domains of environmental health, such as vector control, building design, ventilation, laundry, food safety, and lighting [4]. The WHO’s 2016 infection prevention and control (IPC) guidelines recommend that hand hygiene, cleaning, and waste management are core components of IPC, while also recommending “linkages [with] related programs,” including “WASH” [5]. The 2023 UN Resolution A/78/130 excludes environmental cleaning [1].

Vocabulary shapes roles and responsibilities for achieving and maintaining a safe healthcare environment. “WASH” is often described by its practitioners as an economic sector (e.g., like energy or grid electricity) and has historically focused on community and household-level services. Framing the issue as WASH implies responsibility for a specific set of WASH actors—conventionally from engineering and related disciplines—while deemphasizing the health sector’s critical role in funding, implementing, and regulating environmental health services in healthcare facilities. Differing vocabularies shape different priorities and actions across WASH and health actors, which can create overlapped or competing priorities, impede coordination, and undermine long-term improvement.

Vocabulary influences monitoring environmental health services and related targets and investments. For example, in 2017, the WHO and UNICEF released the Water and Sanitation for Health Facility Improvement Tool (WASH FIT), a risk-based quality improvement tool [6]. The tool aligns with the SDG indicators for water, sanitation, hygiene, waste management, and cleaning. As of 2024, over 70 countries use WASH FIT, which informs national strategies and guides billions of investment dollars. Yet the widespread use of WASH FIT and a broader WASH framing raises the question: Are other domains neglected by framing programs around WASH?

There is no solid evidence base for the narrow emphasis on WASH as water, sanitation, and hygiene. Systematic reviews indicate that hand hygiene reduces healthcare-acquired infections, but evidence on water and sanitation is inconclusive [7]. Other domains, such as building design and cleanliness, directly affect patient experiences through privacy, dignity, care satisfaction, and exposure to pathogens and other physical hazards. However, they are understudied, partly because the vocabulary to define indicators and quantify effects is underdeveloped [8].

Vocabulary rooted in environmental health provides a way forward. Environmental health is a well-established discipline that considers the effects of the natural and built environment on health and well-being. This encompasses domains traditionally included under WASH and others such as ventilation and drainage that are important for health and wellbeing but have been neglected under recent framings [9]. “Environmental health services” provides a better starting point for a descriptor, but this will require discussion and debate.

Efforts must be made to identify the scope and boundaries of specific services falling within environmental health services and define different service levels, from basic to advanced, that will be needed in different facility types and contexts. Some services, such as water and sanitation, already have well-established definitions and indicators that can be straightforwardly incorporated. Others, such as hygiene, cleaning, and waste management, are well accepted within the scope but have competing definitions and indicators in medicine and environmental engineering requiring reconciliation. Services that have been historically included but more recently pushed to the margins of disciplinary bounds need reincorporation (e.g., vector control, ventilation, and food hygiene). Cross-cutting issues, such as gender and climate, should be reflected within this vocabulary. We propose framing the scope around the services in the 2008 WHO report, “Essential Environmental Health Standards in Health Care,” which applies a broad environmental health perspective (Fig 1).

Developing this controlled vocabulary will unite different actors to focus on various components of environmental health for safer healthcare facilities. This will require multi-sectoral discussion and consensus among WASH, medical, health systems, and environmental engineering stakeholders. We expect that replacing current vocabulary across different stakeholders will face some pushback, particularly where considerable investment has been made in developing tools and guidelines around “WASH” from the WHO, UNICEF, and others (e.g., WASH FIT), which have become embedded into practice. Some terms for environmental health services have been established within the medical sector with specific meanings for decades (e.g., standard precautions, infection prevention, and control), which will inevitably resist change. Nevertheless, we argue that a controlled vocabulary is important to galvanize and coordinate action with greater efficiency and impact.

The upcoming 2030 expiration of the SDGs and debate about their replacement offers an entry point for dialog. Reframing this issue in preparation for the post-2030 agenda presents an opportunity to galvanize action around environmental health services that have been historically neglected but will be critical to respond to climate change, water scarcity, pandemics, and emergent challenges threatening future health systems. These steps will be challenging, but we argue they are necessary to coordinate efforts and reach shared goals to protect the health and well-being of patients, healthcare workers, and communities.

## Figures and Tables

**Fig 1.**
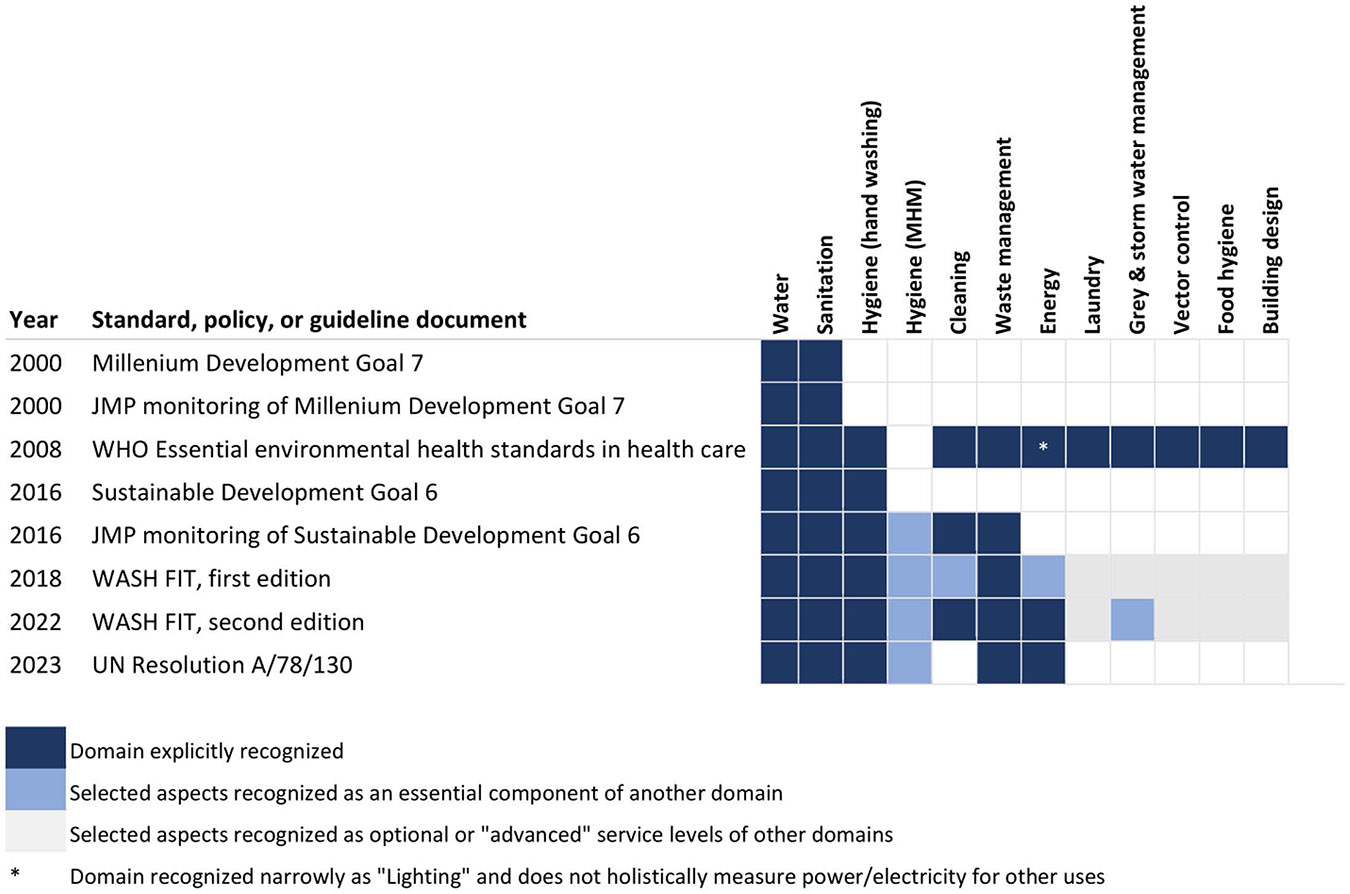
Evolution in scope and vocabulary used to describe environmental health services in various international standards, policies, and guidelines documents. MHM, menstrual hygiene management.
